# Utilization of Molecular, Phenotypic, and Geographical Diversity to Develop Compact Composite Core Collection in the Oilseed Crop, Safflower (*Carthamus tinctorius* L.) through Maximization Strategy

**DOI:** 10.3389/fpls.2016.01554

**Published:** 2016-10-19

**Authors:** Shivendra Kumar, Heena Ambreen, Murali T. Variath, Atmakuri R. Rao, Manu Agarwal, Amar Kumar, Shailendra Goel, Arun Jagannath

**Affiliations:** ^1^Department of Botany, University of DelhiNew Delhi, India; ^2^Centre for Agricultural Bioinformatics, Indian Council of Agricultural Research-Indian Agricultural Statistics Research InstituteNew Delhi, India

**Keywords:** safflower, phenotypic data, AFLP, regional gene pools, Maximization (M) strategy, MSTRAT, POWERCORE, core collection

## Abstract

Safflower (*Carthamus tinctorius* L.) is a dryland oilseed crop yielding high quality edible oil. Previous studies have described significant phenotypic variability in the crop and used geographical distribution and phenotypic trait values to develop core collections. However, the molecular diversity component was lacking in the earlier collections thereby limiting their utility in breeding programs. The present study evaluated the phenotypic variability for 12 agronomically important traits during two growing seasons (2011–12 and 2012–13) in a global reference collection of 531 safflower accessions, assessed earlier by our group for genetic diversity and population structure using AFLP markers. Significant phenotypic variation was observed for all the agronomic traits in the representative collection. Cluster analysis of phenotypic data grouped the accessions into five major clusters. Accessions from the Indian Subcontinent and America harbored maximal phenotypic variability with unique characters for a few traits. MANOVA analysis indicated significant interaction between genotypes and environment for both the seasons. Initially, six independent core collections (CC1–CC6) were developed using molecular marker and phenotypic data for two seasons through POWERCORE and MSTRAT. These collections captured the entire range of trait variability but failed to include complete genetic diversity represented in 19 clusters reported earlier through Bayesian analysis of population structure (BAPS). Therefore, we merged the three POWERCORE core collections (CC1–CC3) to generate a composite core collection, CartC1 and three MSTRAT core collections (CC4–CC6) to generate another composite core collection, CartC2. The mean difference percentage, variance difference percentage, variable rate of coefficient of variance percentage, coincidence rate of range percentage, Shannon's diversity index, and Nei's gene diversity for CartC1 were 11.2, 43.7, 132.4, 93.4, 0.47, and 0.306, respectively while the corresponding values for CartC2 were 9.3, 58.8, 124.6, 95.8, 0.46, and 0.301. Each composite core collection represented the complete range of phenotypic and genetic variability of the crop including 19 BAPS clusters. This is the first report describing development of core collections in safflower using molecular marker data with phenotypic values and geographical distribution. These core collections will facilitate identification of genetic determinants of trait variability and effective utilization of the prevalent diversity in crop improvement programs.

## Introduction

Safflower (*Carthamus tinctorius* L.) is a dryland oilseed crop widely adapted to grow over a broad range of geographical locations extending from Far East to American region (Dajue and Mündel, 1996). It was initially cultivated for extraction of dyes and subsequently gained importance as a source of edible oil due to its nutritionally desirable composition of plant-based unsaturated fatty acids namely, oleic, and linoleic acid (Ashri et al., 1977; Dajue and Mündel, 1996; Khan et al., 2009). In addition, the medicinal properties of safflower and its use as a system for production of pharmaceutical products are well documented (Weiss, 1983; McPherson et al., 2009; Carlsson et al., 2014). Safflower is severely affected by several biotic and abiotic stresses and is characterized by low yield and spiny nature which have discouraged farmers from adopting its cultivation in several countries including India (Nimbkar, 2008). Moreover, the breeding lines and cultivars of safflower harbor low genetic diversity (Kumar et al., 2015), which restricts their utility in breeding programs. Therefore, an extensive characterization of the prevalent genetic and phenotypic diversity among the global germplasm of the crop is required to facilitate development of effective crop improvement strategies.

Germplasm resources act as a reservoir for trait variability and are of prime importance for crop improvement. However, their large size and heterogeneous structure restricts their accessibility and application (Brown, 1989a,b; Noirot et al., 1996; van Hintum, 2000). For effective management and utilization of these resources, Frankel (1984) introduced the concept of “core collection.” A core collection is a representative subset of minimum number of non-redundant individuals capturing maximum variability prevalent in the entire germplasm collection. Characterization and evaluation of core collection is an easier task compared to the entire germplasm collection. Initially, core collections were developed using morphological parameters and/or geographical distribution (Huaman et al., 1999; Tai and Miller, 2001; Upadhyaya and Ortiz, 2001; Upadhyaya et al., 2003, 2009; Li et al., 2005; Bhattacharjee et al., 2007; Mahalakshmi et al., 2007). Subsequently, availability of molecular markers and their greater efficacy in elucidating genetic diversity have facilitated the development of more robust core collections using molecular markers either alone (Zhang et al., 2009) or in conjunction with phenotypic data in various crop species (Wang et al., 2006; Ebana et al., 2008; Shehzad et al., 2009; Belaj et al., 2012; Díez et al., 2012; Liu et al., 2015).

Until now, efforts to consolidate safflower genetic resources into core collections were based on assessment of morphological traits and geographical distribution. Johnson et al. (1993) developed the first core collection in safflower consisting of 210 accessions by evaluating a germplasm collection of 2042 accessions from ~50 countries. Dwivedi et al. (2005) developed another core collection comprising 570 accessions from a total collection of 5522 safflower accessions from 38 countries. However, since most agronomically important traits are quantitative in nature, they are significantly influenced by genotype × environment (GE) interactions. Therefore, the data types (morphological and geographical information) used for development of the initial core collections in safflower would have under-represented the genetic diversity present in the crop due to lack of allelic information. Efforts are required to include genetic diversity based on molecular markers for development of a more effective and robust core collection in safflower.

The present study describes the phenotypic evaluation of a global representative collection of 531 safflower accessions and development of a robust core collection in safflower using maximization strategy. To the best of our knowledge, this is the first report of a composite core collection in safflower utilizing molecular variability along with geographical distribution and phenotypic data. This collection will be useful in designing crop improvement programs in a more effective manner and in dissecting the molecular determinants of trait variability.

## Materials and methods

### Germplasm resources

The safflower germplasm used in the present study comprised of 531 accessions. The details of the accessions including their PI numbers, country of origin and regional pool along with the strategy used for their selection has been described by Kumar et al. (2015).

### Measurement of phenotypic data

The accessions were grown and characterized in two consecutive seasons (2011–12 and 2012–13) at Agricultural Research Station, University of Delhi, Bawana Road, New Delhi, India (Latitude: 28° 38′ N, longitude: 77° 12′ E and altitude: 252 m). Ten seeds of each accession were sown in a single row of 2 m with an average distance of 0.2 m between plants and a gap of 0.6 m between each row. Locally adopted agronomic practices were followed for raising a healthy crop.

Phenotypic characterization was done following the guidelines of International Plant Genetic Resources Institute (IPGRI) for safflower. Each accession was characterized for 12 traits which included 8 pre-harvest and 4 post-harvest traits. The pre-harvest traits were growth habit (GH), plant height (PH), spininess (SP), number of primary branches (PB), branch location (BL), number of heads per plant (HD), flower color (FC), and days to 50% flowering (DTF). The post-harvest traits were 100-seed weight (SW), seed oil content (OC), oleic acid content (OA), and linoleic acid content (LA). The data was recorded for three healthy plants of each accession.

Growth habit of the plant was recorded as “erect” or “sprawling” on ground. For plant height, main shoot length was measured from soil surface to the highest inflorescence of the plant. Spininess of the accessions were recorded at the onset of flowering and reported as “present” or “absent.” Number of branches originating from the main axis was counted as number of primary branches. Distribution of primary branches on the main shoot determined branch location in safflower and was categorized as basal, upper one-third, upper two third, and from base to apex of the plant. The total number of inflorescences (primary, secondary, and tertiary) per plant was recorded as number of heads per plant. Flower color was documented as yellow, orange, red and off-white at full bloom stage. For each accession, the number of days from planting to onset of flowering in 50% plants was considered as days to 50% flowering. Seed weight of 100 achenes from each plant was measured in grams and recorded as 100-seed weight. Oil content was measured by Near-Infrared Reflectance Spectroscopy (NIRS) (Foss, Germany). Oil content in seed samples of 300 safflower accessions was estimated by Soxhlet method and used for the development and calibration of NIRS equations for oil content measurement in safflower (manuscript under preparation). Fatty acid composition (oleic and linoleic acid content), was determined by methyl esterification followed by gas chromatography using *Clarus* 580 (Perkin Elmer, USA) as per manufacturer's instructions.

### Statistical analysis of phenotypic data

Phenotypic correlations between different quantitative traits (computed as Pearson correlation coefficient, r), cluster analysis based on Euclidean distance and two-dimensional Principal coordinate analysis (PCoA) were performed using PAST *version* 3.10 (Hammer et al., 2001). Frequency distribution of accessions for different classes of traits was calculated. Evaluation of seasonal variation for the traits under consideration was conducted through Multivariate Analysis of Variance (MANOVA) using SPSS version 18 (Statistical Package for the Social Sciences; SPSS Inc. Released, 2009. PASW Statistics for Windows, Version 18.0. Chicago: SPSS Inc.).

### Development of core collections

MSTRAT (Gouesnard et al., 2001) and POWERCORE (Kim et al., 2007) were used for development of independent core collections using phenotypic data of seasons 2011–12, 2012–13 and genotypic data reported by Kumar et al. (2015). In MSTRAT, 20 replicates and 100 iterations were tested at a fixed sample size of 10%. The core collection with highest Shannon's diversity index was selected. POWERCORE was used as described in the user's manual (Kim et al., 2007).

### Evaluation of core collections

Core collections were evaluated by estimating Shannon's diversity index (*I*) and Nei's gene diversity (*H*) using POPGENE version 1.32 (Yeh et al., 1999). Additionally, *mean difference percentage* (MD%), *variance difference percentage* (VD%), *variable rate of coefficient of variance* (VR%), and *coincidence rate of range* (CR%) were calculated to assess the level of diversity captured in core collection with respect to the entire collection (Hu et al., 2000). *T*-test and *F*-test were performed to study difference in mean and variance of traits between the entire collection and composite core collections. The “*coverage*” criterion described by Kim et al. (2007) was used to evaluate the percentage diversity captured for each variable in the composite core collections.

## Results

### Analysis of pre-harvest traits

Analysis of pre-harvest traits revealed significant phenotypic variability among the safflower accessions used in the current study. Erect growth was observed in 529 accessions while two accessions (PI-305204 and PI-306912) showed sprawling growth in both the seasons (2011–12 and 2012–13). Plant height of the studied accessions ranged from 94 to 226 cm in 2011–12 and from 73 to 211 cm in 2012–13 growing seasons (Supplementary Figures 1A,B). Although these values suggest a minor shift in the overall range between the two seasons, plant height of individual accessions did not show a markable difference. In our study, around 21% of the accessions (111) were non-spiny while 79% of accessions (420) were spiny in nature. The number of primary branches in the studied accessions ranged from 4 to 34 in 2011–12 season and from 5 to 33 for 2012–13 season. The position of branch emergence is associated with the bushy nature in safflower. A large number of accessions (38%) had branches located in the upper one third portion of the plant followed by 31% of accessions with branches in the upper two third portion. The remaining 31% of accessions had branches originating from the base till the apex giving it a more bushy appearance.

The number of heads per plant varied from 11 to 203 and from 9 to 189 for 2011–12 and 2012–13 growing seasons, respectively. Safflower shows different shades for its corolla color varying from yellow, orange, red to off-white. In our study, yellow was the most common color (76% of accessions) followed by orange (11% of accessions). Days to 50% flowering was recorded for each accession as described above. The trait distribution was observed to be asymptotically normal in both the seasons (Supplementary Figures 1C,D). Based on these observations, accessions were categorized as early flowering (tail of the distribution curve; 119–128 days and 137–146 days for 2011–12 and 2012–13 seasons, respectively), mid flowering (129–151 days for 2011–12 and 147–174 days for 2012–13, respectively) and late flowering (tail of the distribution curve; 152–160 days and 175–182 days for 2011–12 and 2012–13 season, respectively; Supplementary Figures 1C,D). Although days to 50% flowering shifted between the two seasons, no change was observed in the associated categories of accessions between the seasons. Based on the above analysis, we identified 14 early-flowering, 490 mid-flowering, and 27 late-flowering accessions.

### Analysis of post-harvest traits

The hundred seed weight value ranged from 1 to 8 g for 2011–12 season and from 2 to 8 g in 2012–13 season. No significant difference was observed in the phenotypic range between the two seasons. Estimation of oil content was performed using NIRS. The oil content among the analyzed accessions ranged from 16 to 50% in 2011–12 while for the 2012–13 season it ranged from 15 to 47% (Supplementary Figures 1E,F). Accessions with oil content < 22% were considered as “low oil content” while those with >40% oil content were categorized as “high oil content” (Supplementary Figures 1E,F). Accessions with low and high oil content remained consistent in both the seasons. The oleic acid content ranged from 9 to 82% with most accessions (93%) falling in the lower range of oleic acid content (below 25%) and a few (7%) having medium and high oleic acid content (>75%). Linoleic acid content varied from 13 to 87% with most accessions (90%) showing high linoleic acid content (65–80%) and few accessions having very high (3%), medium or low linoleic acid content (6.6%). Table 1 includes list of accessions with high oil content (>40%), high oleic acid (>75%), and very high linoleic acid (≥80%) observed in the current study.

**Table 1:** **List of safflower accessions with high oil content (>40%), linoleic acid content (≥80%), and oleic acid content (>75%)**.

**Oil content (>40%)**			**Linoleic acid (≥80%)**			**High Oleic acid (>75%)**		
**PI number**	**Country of origin**	**Oil content (%)**	**PI number**	**Country of origin**	**Linoleic acid content (%)**	**PI number**	**Country of origin**	**Oleic acid content (%)**
537635	USA	50	250081	Egypt	87	613394	USA	82
537701	USA	48	544025	China	82	560177	USA	81
560169	USA	47	560188	USA	81	560165	USA	81
537662	USA	46	305198	India	81	560173	USA	81
560175	USA	45	543992	China	81	560166	USA	80
560172	USA	45	514624	China	80	560169	USA	79
560168	USA	43	613459	Portugal	80	401474	Bangladesh	78
537693	USA	43	537654	USA	80	560172	USA	77
537110	USA	43	560185	USA	80	560168	USA	77
560177	USA	42	560176	USA	80	401589	India	77
537677	USA	42	537645	USA	80	537712	USA	77
560171	USA	42	537653	USA	80	470942	Bangladesh	77
537696	USA	41	251987	Turkey	80	401470	Bangladesh	76
537656	USA	41	426186	Afghanistan	80	401477	Bangladesh	76
537645	USA	41	306685	Israel	80	401476	Bangladesh	76
–	–	–	–	–	–	401479	Bangladesh	76

### Correlation analysis between traits

Correlation analysis indicated a significant negative correlation (*r* = −0.99) between oleic and linoleic acid content of safflower. The correlation values for all other traits were below the significance level of 0.50. The highest positive correlation value was observed between number of heads per plant and number of primary branches (0.45). The correlation coefficient values for the analyzed traits are listed in Table 2.

**Table 2:** **Correlation coefficient between eight quantitative traits studied in the entire safflower collection**.

	**OC**	**OA**	**LA**	**SW**	**PH**	**NH**	**NB**	**DTF**
OC	^*^							
OA	0.133	^*^						
LA	−0.142	−0.996	^*^					
SW	0.044	−0.088	0.094	^*^				
PH	−0.130	−0.122	0.120	−0.185	^*^			
NH	0.065	−0.049	0.039	0.077	0.005	^*^		
NB	0.109	0.024	−0.028	−0.039	0.134	0.450	^*^	
DTF	0.164	0.027	−0.033	−0.053	0.098	0.033	0.090	^*^

*OC, oil content; OA, oleic acid content; LA, linoleic acid content; SW, 100-seed weight; PH, plant height; NH, number of heads per plant; NB, number of primary branches; DTF, days to 50% flowering*.

* *Denotes correlation between same trait*.

### Distribution of traits within and between regional gene pools

The 531 accessions used in this study represented all the 10 regional gene pools defined by Ashri (1975) based on morphological parameters. The distribution of different phenotypic classes among the safflower regional gene pools is given in Supplementary File 1. Although morphological delineation was not prominently observed between different regional gene pools for most traits, a few character states were more pronounced in some gene pools. Accessions with increased plant height (>155 cm) were limited to Iran-Afghanistan, Turkey, Far East, and Europe. The majority of accessions with low head count per plant were from the Far East. A higher number of primary branches (25–33) was found only among accessions from the Indian subcontinent, Far East, America, and Iran-Afghanistan. Early flowering accessions were found only among genotypes from Far East, Indian subcontinent, Egypt, and America. On the other hand, all other pre-harvest traits namely growth habit, spines, location of branches on the main axis of plant and flower color did not show any preferential distribution to any regional gene pool.

Among post-harvest traits, high oil content was observed only in accessions from the American region while some accessions from the Indian subcontinent had up to 40% of oil content (Supplementary File 1). High oleic acid content (>75%) was found only in accessions from America and Indian subcontinent. All accessions from Near East, Turkey, Egypt, Sudan, Europe, and Iran-Afghanistan had low oleic acid content. Higher ranges of 100 seed weight (6–8 gm) were found predominantly among Indian accessions and to a limited extent from American region.

### Cluster analysis and principal coordinate analysis (PCoA)

**Figure 1:**
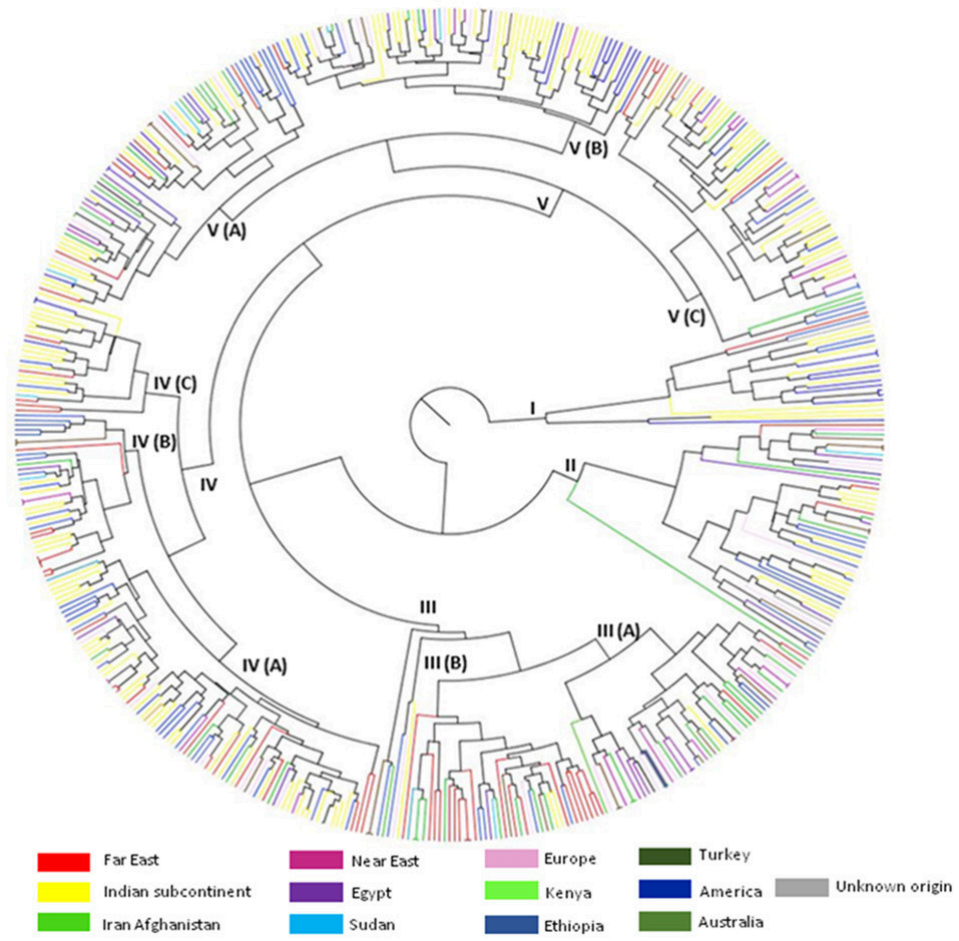
**UPGMA cluster analysis illustrating the genetic relationships between 531 safflower accessions based on 12 morphological traits**. Five clusters (I–V) with internal sub-groupings are shown. Color codes correspond to region of origin.

The inter-relationships and genetic distance between safflower accessions based on phenotypic data was assessed through unweighted pair group method with arithmetic mean (UPGMA) clustering using Euclidean distance matrix (Figure 1). Safflower accessions were grouped in five major clusters designated as CL I–CL V. Information on distribution of accessions in different clusters is given in Table 3. CL V is the largest cluster with 215 accessions. All clusters, except Cluster III, were dominated by accessions from the Indian subcontinent and America. Cluster III had significant representation of accessions from Iran-Afghanistan, Far-East, and Europe.

**Table 3:** **Distribution of accessions from different regional gene pools of safflower in clusters of UPGMA dendrogram constructed using phenotypic data**.


	** 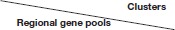 **	**CL I**	**CL II**	**CL III**		**CL IV**			**CL V**		
				**III A**	**III B**	**IV A**	**IV B**	**IV C**	**V A**	**V B**	**V C**
1.	Far East (FE)	1	3	3	20	12	7	5	7	1	7
2.	Indian subcontinent (IS)	8	12	1	3	34	8	14	15	41	30
3.	Iran-Afghanistan (IA)	−	5	15	9	6	1	−	11	4	4
4.	Near East (NE)	−	1	8	−	3	2	−	2	3	10
5.	Turkey (TU)	−	6	7	4	1	2	−	2	3	1
6.	Egypt (EG)	−	5	6	1	2	1	−	3	2	1
7.	Sudan (SU)	−	1	−	2	−	1	1	3	2	−
8.	Kenya (KE)	−	−	−	−	−	−	−	1	−	−
9.	Ethiopia (ET)	−	−	−	1	1	−	−	−	−	
10.	Europe (EU)	−	4	8	2	8	−	−	6	7	2
11.	America (US)	13	12	4	6	17	11	5	17	16	11
12.	Australia (AUS)	−	−	−	−	1	−	−	1	1	
13.	Unknown origin (UN)	1	1	−	−	−	−	−	−	−	1
	Total number of genotypes	23	50	52	48	85	33	25	68	80	67

**Figure 2:**
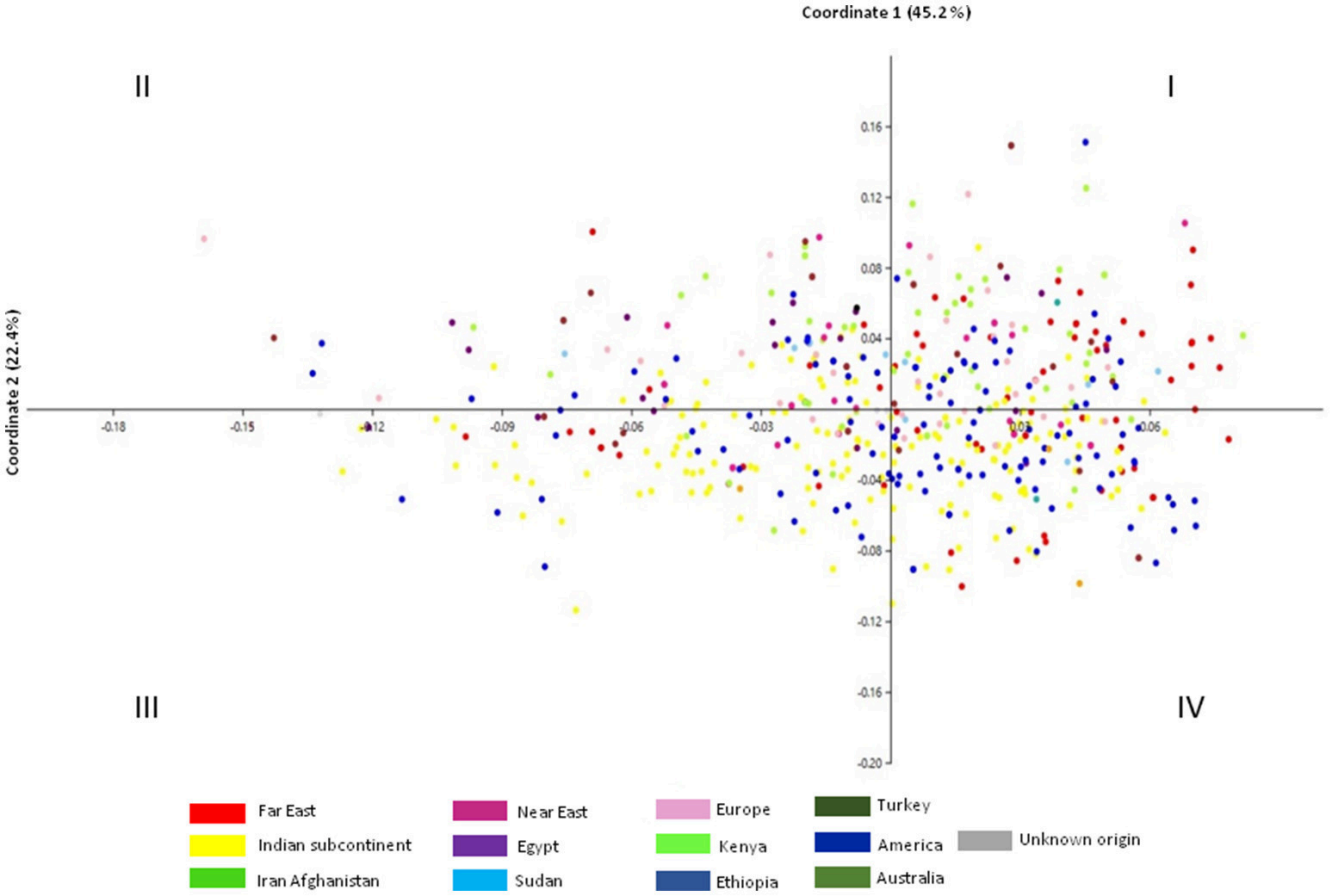
**Principal coordinate analysis of 531 safflower accessions based on Euclidean distance matrix using 12 morphological traits**. Color codes correspond to region of origin.

In principal coordinate analysis (PCoA), coordinate axes 1 and 2 captured 42.5 and 22.4%, respectively of the total existing variation among the accessions (Figure 2). Accessions from Indian subcontinent were mainly present in quadrants III and IV with minor representation in quadrants I and II. Accessions from American region were homogenously distributed among all the quadrants of PCoA obtained using phenotypic data. Accessions from Iran-Afghanistan region were mainly found in quadrants I and II with a few accessions in quadrants III and IV. Far East accessions were restricted to quadrants I and IV while accessions from the European region were limited to quadrant I and II. Accessions from the Near East region were found to be part of quadrants I and II while Sudanese accessions were distributed in all the four quadrants. Accessions from Turkey and Egypt were predominantly found in quadrants I and II.

### Analysis of seasonal variations and development of core collections using POWERCORE and MSTRAT

MANOVA analysis indicated significant seasonal effects as well as significant interaction effect between seasons and accession effects by considering all quantitative traits together (Table 4). Therefore, phenotypic data for both the seasons (2011–12 and 2012–13) and molecular marker data were treated independently for development of core collections. Usage of the two maximization (M) strategy based programs resulted in the generation of six core collections (CC1-CC6).

**Table 4:** **Multivariate analysis of variance (MANOVA) to study seasonal differences in quantitative traits**.

**Effect**	**Value**	***F***	**Sig**.
Accession	0.001	22.807	< 0.001
Season	0.209	1860.796	< 0.001
Replication	0.966	8.541	< 0.001
Accession ^*^ Season	0.104	3.306	< 0.001

Based on Wilks' Lambda.

In our earlier work, molecular profiling of the 531 accessions identified 157 polymorphic AFLP markers (Kumar et al., 2015). Core collections were developed with these AFLP markers using POWERCORE and MSTRAT and designated as CC1 and CC4, respectively. CC1 included 14 accessions (2.6% of the entire collection) belonging to six out of 10 regional gene pools while CC4 comprised 26 accessions (4.9% of the entire collection) belonging to seven regional gene pools (Table 5). Phenotypic data of seasons 2011–12 and 2012–13 was used to develop core collections CC2 and CC3, respectively using POWERCORE. CC2 consisted of 26 accessions (4.9% of the entire collection) from six regional gene pools (Table 5) and regions of secondary introduction (Australia and America). CC3 consisted of 27 accessions (5.1% of the entire collection) from six regional gene pools of safflower. Core collections CC5 and CC6, were developed using phenotypic data of season 2011–12 and 2012–13, respectively using MSTRAT. CC5 consisted of 47 accessions (8.8% of the entire collection) from seven regional gene pools and regions of secondary introduction (America and Australia). CC6, comprising 54 accessions (10% of the entire collection) had representation from eight regional gene pools along with regions of secondary introduction.

**Table 5:** **Representation from different regional gene pools of safflower in developed core collections (CC)**.

**Regional gene pools**	**Entire collection**	**POWERCORE**			**MSTRAT**			**Composite core collections**	
		**CC1**	**CC2**	**CC3**	**CC4**	**CC5**	**CC6**	**CartC1**	**CartC2**
Far East (FE)	67	2	4	4	2	7	9	7	14
Indian subcontinent (IS)	167	5	5	8	7	12	13	14	26
Iran-Afghanistan (IA)	54	1	3	0	4	5	7	4	13
Near East (NE)	26	0	2	1	3	2	0	3	5
Turkey (TU)	29	1	1	0	3	0	1	2	4
Egypt (EG)	20	1	2	2	0	4	3	4	5
Sudan (SU)	10	0	0	0	1	0	0	0	1
Kenya (KE)	1	0	0	0	0	0	1	0	1
Ethiopia (ET)	2	0	0	1	0	1	1	1	1
Europe (EU)	37	2	2	3	2	2	5	7	7
America (US)	112	2	6	7	4	13	12	14	27
Australia (AUS)	3	0	1	1	0	1	1	1	1
Unknown origin (UN)	3	0	0	0	0	0	1	0	1
TOTAL	531	14	26	27	26	47	54	57	106

The ranges, means, and variances for all the quantitative traits were calculated for core collections developed using phenotypic data (CC2, CC3, CC5, and CC6) and compared with corresponding values for the entire collection (Supplementary Tables 1, 2). MD% displays the difference in averages between the core and the entire collection and should be < 20% for a representative core collection. MD% ranged from 6.36 to 15.45% for the four core collections (Table 6). VD% indicates the variance captured by a core collection and ranged from 36.4 to 59% in the current analysis. The coefficient of variance (VR%) captured in the core collection should have a value higher than 100%. CC5 and CC6 had high VR% above 105% while CC2 and CC3 showed a value of ~96.1% (Table 6). The range distribution of traits in a core collection in comparison to entire collection is measured by CR% whose value should be greater than 80%. All the analyzed core collections displayed high CR% value ranging from 94.25 to 143.52%. Shannon-Weaver diversity index (*I*) was calculated for all the core collections and ranged from 0.44 to 0.53. The core collections, CC1 and CC4 derived using molecular marker data, showed highest Shannon-Weaver diversity index with a value of 0.53 and 0.49, respectively which was higher than the corresponding values obtained for core collections derived using phenotypic data (Table 6). Nei's genetic diversity (*H*) for the six core collections ranged from 0.273 to 0.346. Similar to Shannon's diversity index, the highest value of *H* was recorded for CC1 (0.346) and CC4 (0.318) (Table 6).

**Table 6:** **Evaluation indices for developed core collections**.

**Core Collection**	**MD%**	**VD%**	**VR%**	**CR%**	***I***	***H***
CC 1	1.38	12.4	106.55	100	0.52	0.346
CC 2	13.49	54.64	96.14	143.52	0.45	0.293
CC 3	11.03	49.81	96.1	136.42	0.42	0.273
CC 4	3.8	18.76	95.5	66.5	0.48	0.318
CC 5	6.36	36.4	116.8	94.25	0.44	0.289
CC 6	15.45	59	147.25	95.75	0.44	0.289
CartC1	11.2	43.7	132.4	93.4	0.47	0.306
CartC2	9.3	58.8	124.6	95.8	0.46	0.301

MD%, mean difference; VD%, variance difference; VR%, variable rate; CR%, coincidence rate of range; I, Shannon's diversity index, and H, Nei's genetic diversity.

### Development and evaluation of composite core collections

**Figure 3:**
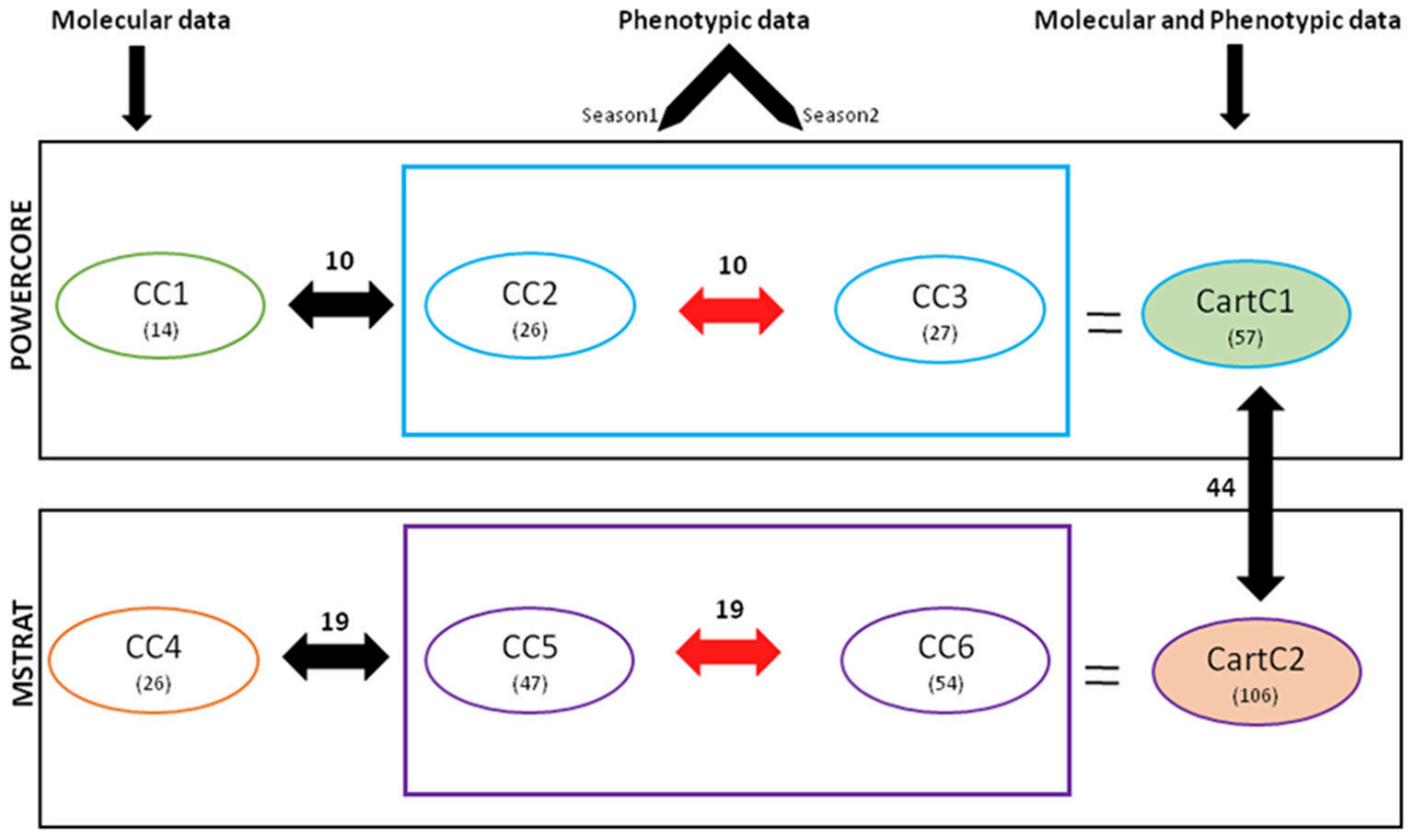
**Flowchart describing the strategy and results of development of core collection for safflower**. Numerical values in parenthesis indicate the number of accessions in respective cores. Values indicated above the double-headed arrows depict the number of accessions common between different core collections.

Based on the various indices described above, all the core collections developed in our study appeared to represent the prevalent diversity of the entire collection. However, none of the core collections contained representation from all the 19 clusters derived by Bayesian Analysis of Population Structure (BAPS) (Table 7), which captured diverse combinations of alleles and resulted in meaningful genetic stratification of the collection (Kumar et al., 2015). In order to capture the maximum range of allelic diversity/trait state in a core collection and prevent trade-off between two data types when used together, we attempted to combine phenotypic and molecular variability by merging core collections derived from each strategy separately (Figure 3). The core collections developed by POWERCORE, i.e., CC1 (14 accessions), CC2 (26 accessions), and CC3 (27 accessions) were combined to form a non-redundant composite core collection referred to as CartC1 (Supplementary File 2). CartC1 comprised 57 accessions (10.7% of initial collection) representing 19 BAPS clusters, eight regional gene pools and two regions of secondary introduction for safflower (Tables 5, 7). Similarly, the core collections derived through MSTRAT, i.e., CC4 (26 accessions), CC5 (47 accessions), and CC6 (54 accessions) were merged resulting in a non-redundant composite core collection referred to as CartC2 (Supplementary File 2). CartC2 consisted of 106 accessions (~20% of initial collection) including representation from all 19 BAPS clusters, ten regional gene pools and two regions of secondary introduction for safflower (Tables 5, 7). Forty four accessions were common among the two composite core collections (Figure 3).

**Table 7:** **Distribution of accessions of developed core collections in different BAPS clusters derived based on AFLP markers by Kumar et al. (2015)**.

**BAPS clusters**	**Number of accessions**							
	**CC1**	**CC2**	**CC3**	**CC4**	**CC5**	**CC6**	**CartC1**	**CartC2**
Bacl 1	−	1	1	−	3	2	2	4
Bacl 2	2	1	1	2	1	3	4	5
Bacl 3	−	1	1	3	2	6	1	7
Bacl 4	1	1	1	3	2	2	2	6
Bacl 5	1	3	2	−	5	5	5	7
Bacl 6	1	−	−	2	−	2	1	4
Bacl 7	1	1	3	2	5	3	6	8
Bacl 8	1	1	−	1	−	1	2	2
Bacl 9	1	2	2	1	3	3	2	6
Bacl 10	−	1	2	−	4	4	2	6
Bacl 11	1	1	−	2	1	2	2	5
Bacl 12	1	3	−	3	4	2	4	8
Bacl 13	−	2	2	−	1	3	4	7
Bacl 14	1	3	4	2	5	5	8	12
Bacl 15	−	1	1	−	1	2	1	2
Bacl 16	1	1	4	−	2	4	5	4
Bacl 17	−	1	1	2	1	1	1	4
Bacl 18	1	1	1	2	3	4	2	8
Bacl 19	1	1	1	1	−	−	3	1

The ranges, means and variances for all the quantitative traits for CartC1 and CartC2 are provided in Table 8. Homogeneity tests were performed to evaluate the difference in means (*t*-test) and variances (*F*-test) of traits between the entire collection and composite core collections (α = 0.05; Table 8). For a core collection to be representative of the entire collection, it is expected that the difference in mean should not deviate by more than 20% for the traits (Hu et al., 2000). Difference between the mean of the entire collection and CartC1 was non-significant for oil content, 100 seed weight, plant height, number of heads per plant, number of primary branches per plant, and days to 50% flowering. We observed non-significant differences in variance for three traits (100 seed weight, plant height, number of primary branches per plant) between CartC1 and the entire collection (Table 8). *T*-test provided non-significant differences for oil content, 100 seed weight, plant height, number of heads per plant, and days to 50% flowering while *F*-test revealed non-significant variance for only two traits (100 seed weight and plant height) between CartC2 and the entire collection. In “*Coverage”* analysis (Kim et al., 2007), CartC1 and CartC2 showed 100% *coverage* value for different phenotypic and genetic variables under consideration.

**Table 8:** **Ranges, means, and variances for the entire collection and composite core collections, CartC1 and CartC2**.

Phenotypic Traits^*^	**Entire collection^#^**			**CartC1 (POWERCORE)**					**CartC2 (MSTRAT)**				
	**Range**	**Mean**	**Variance**	**Range**	**Mean**	**Variance**	***T*-test**	***F*-test**	**Range**	**Mean**	**Variance**	***T*-test**	***F*-test**
OC (%)	16–50	31	20.2	18–50	31.4	35.3	NS	S	16–50	32	32.7	NS	S
OA (%)	9–82	19	161.2	10–79	28.9	503.1	S	S	10–79	24	359.0	S	S
LA (%)	13–87	71	151.7	13–87	61.7	476.8	S	S	13–87	67	344.3	S	S
SW (g)	1–8	5	2.0	1–8	4.3	2.2	NS	NS	1–8	4	2.4	NS	NS
PH (cm)	94–226	154	578.3	109–226	155	712.1	NS	NS	94–226	156	735.9	NS	NS
NH	11–203	74	1508	16–203	84	3382	NS	S	16–203	79	2330.8	NS	S
NB	5–33	14	25.5	6–33	16	47.5	NS	NS	6–33	16	40.1	S	S
DTF	119–160	141	44.0	120–160	141	75.4	NS	S	119–160	141	91.0	NS	S

* Abbreviations for phenotypic traits provided in footnote of Table 2;

# *Data for season 2011–2012 presented*.

**Figure 4:**
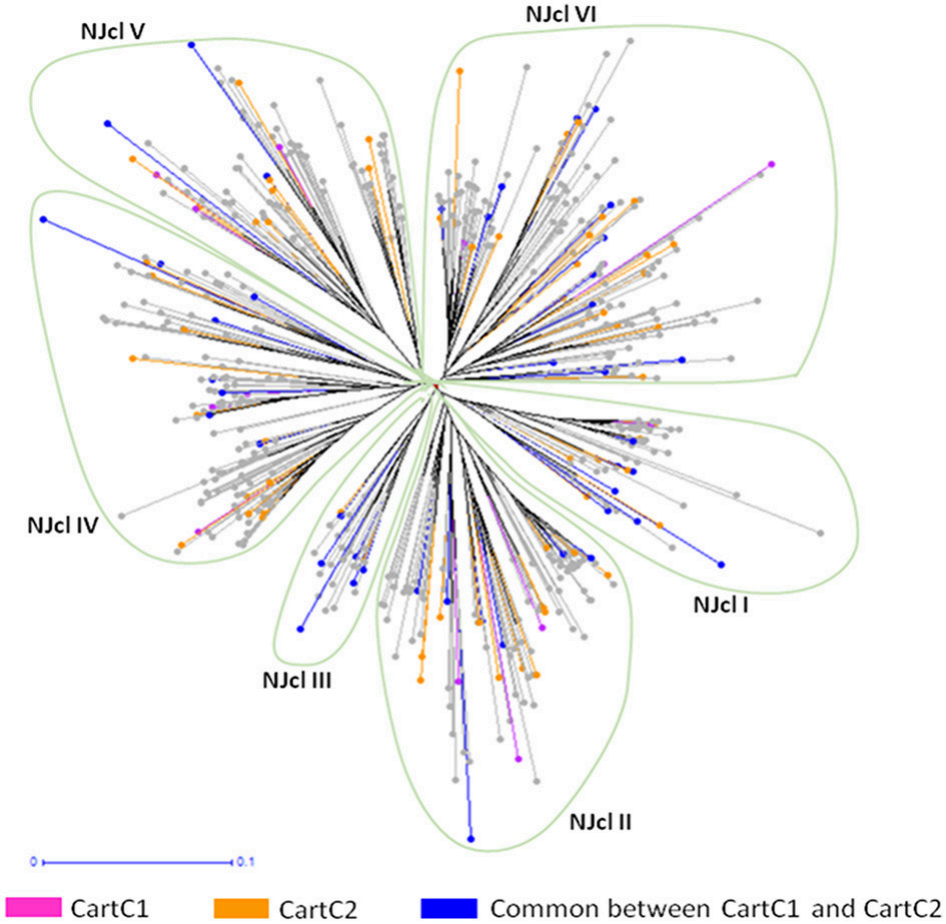
**Distribution of accessions of composite core collections (CartC1 and CartC2) in different clusters of Neighbor joining dendrogram (molecular data)**. Six clusters (NJcl I–VI) are shown. Accessions unique to CartC1 and CartC2 are represented by pink and orange color, respectively. Accessions common between these two collections are represented by blue.

**Figure 5:**
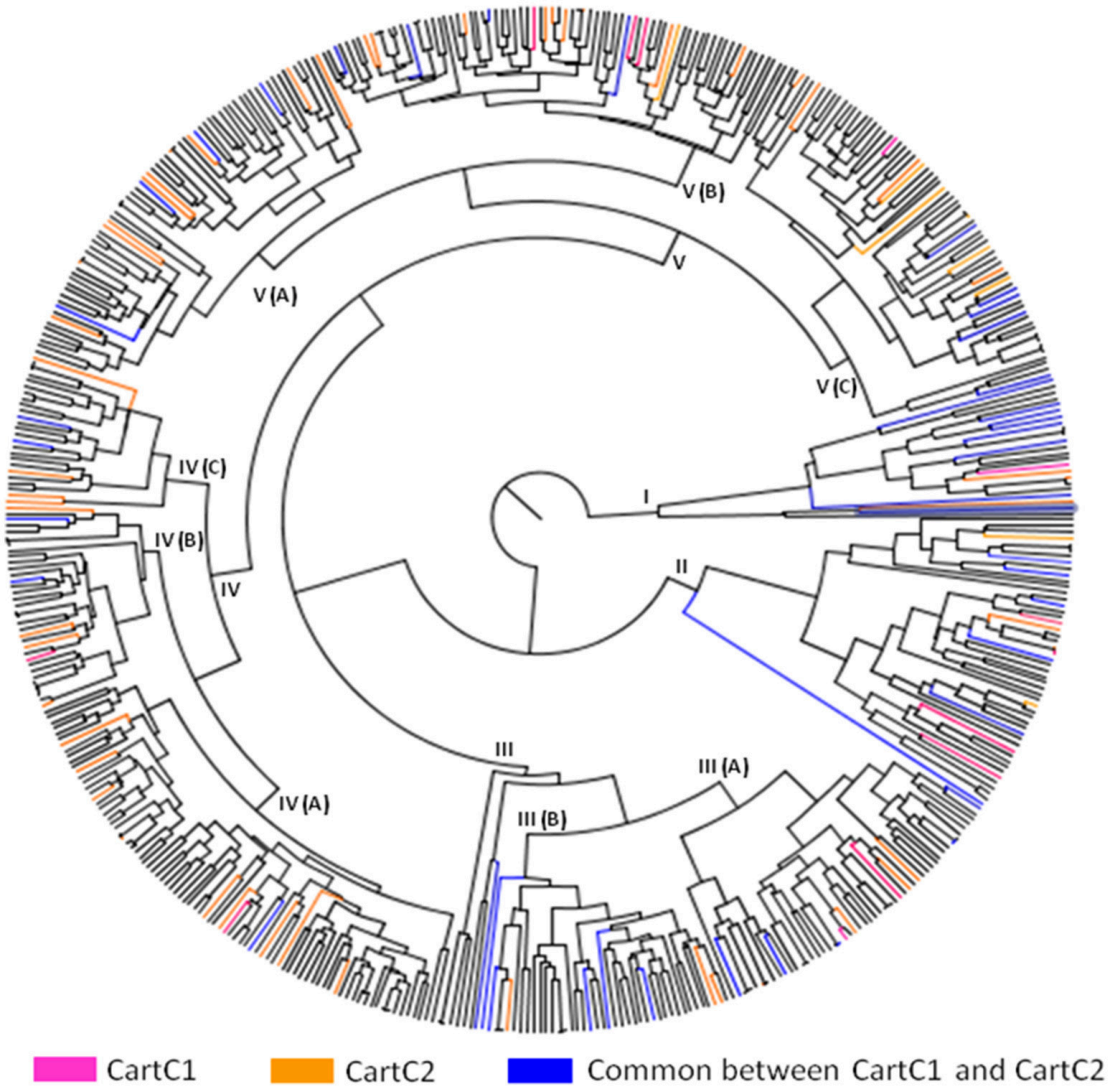
**Distribution of accessions of composite core collections (CartC1 and CartC2) in different clusters of UPGMA dendrogram (morphological data)**. Accessions unique to CartC1 and CartC2 are represented by pink and orange color, respectively. Accessions common between these two collections are represented by blue.

The composite core collections were validated for their representativeness of the entire collection through evaluation indices which are given in Table 6. The Shannon's diversity index (*I*) and Nei's genetic diversity (*H*) were 0.47 and 0.306, respectively for CartC1 and 0.46 and 0.301, respectively for CartC2. We assessed distribution of accessions of CartC1 and CartC2 in the dendrogram obtained through phenotypic and genetic analysis of entire collection. CartC1 and CartC2 showed balanced distribution in all the clusters of Neighbor Joining (genetic analysis; Figure 4) and UPGMA (phenotypic analysis; Figure 5). Thus, CartC1 and CartC2 provided a more rational and exhaustive representation of all the phenotypic and genetic variability than the independent core collections (CC1–CC6) developed in the present study.

## Discussion

A vast collection consisting of 25,179 accessions of safflower is available in 22 gene banks of 15 countries around the world (Zhang and Johnson, 1999). Phenotypic characterization of safflower germplasm in earlier studies demonstrated significant variability for several agronomic traits (Knowles, 1969; Ashri, 1975; Johnson et al., 2001; Amini et al., 2008; Khan et al., 2009). In spite of substantial diversity in its germplasm, yield enhancement in the crop has achieved limited success. Breeding strategies often focus on a limited set of agronomic traits resulting in cultivars with a narrow genetic base. For example, Kumar et al. (2015) showed that the cultivars and breeding lines of safflower from the Indian subcontinent have a narrow genetic base although extensive genetic diversity was present in the regional germplasm. This makes the cultivars highly susceptible to environmental changes and vulnerable to yield penalties. One of the main limitations of earlier approaches has been the over-dependence on morphological and geographical parameters due to lack of information on the genetic structure of safflower germplasm based on molecular markers. The present study attempted to address the above issue by generating two composite core collections in safflower that include data on molecular variability of the crop in addition to phenotypic and geographical parameters.

### Phenotypic diversity of the crop and identification of accessions with desirable agronomic traits

Significant variation was observed among the 531 accessions for 12 agronomic traits. More than 85% of accessions had plant height < 155 cm, which is desirable due to ease of mechanical harvesting from shorter plants (Weiss, 1983). Most safflower varieties and genotypes grown around the world have spines on the leaves and bracts of the plant (Dajue and Mündel, 1996). Spiny nature of the crop is one of the factors responsible for reluctance of farmers to grow safflower, especially in countries like India where harvesting is done manually. Spiny types were widely represented in our collection of 531 accessions. It was hypothesized that non-spiny varieties are generally low in yield and oil content (Dajue and Mündel, 1996). However, we did not observe a significant association between presence of spines and seed oil content. We identified 15 spiny accessions with high seed oil content (Table 1) and several spiny accessions with low seed oil content in the representative collection. The genetics of oil content and spines needs to be investigated further in order to design effective breeding strategies involving these traits.

Traits such as number of primary branches and heads per plant influence seed yield (Ashri et al., 1974; Patil et al., 1994; Dajue and Mündel, 1996). We found significant variation in the above traits and accessions with high number of primary branches and increased number of heads were identified. Analysis of seed yield for these accessions is required to identify promising genotypes. Days to 50% flowering varied between the two growing seasons and ranged from 119 to 160 days (in 2011–12) and from 137 to 182 days (in 2012–13). Delayed flowering in the second year was attributable to cooler temperatures in February and March than in the previous year. The average maximum temperature recorded for the months of February and March 2012 was ~29°C while the corresponding value was ~23°C in 2013. (http://www.weatherspark.com). Though temperature fluctuations did affect developmental stages as well as flowering-related events, the early flowering accessions were consistent between the two seasons.

Identification and use of high oil yielding genotypes is important for increasing oil content in safflower cultivars. Breeding efforts in America led to the development of cultivars with increased seed oil content ranging from 45 to 55% (Bergman et al., 1985; Rubis et al., 2001). However, such improvements are lacking among Indian cultivars which have oil content ranging from 27 to 35%. Evaluation of oil quantity in the 531 accessions by NIRS identified 15 accessions with high oil content (>40%). These would serve as important breeding material in safflower. All the high oil yielding accessions (>40%) had low 100-seed weight (3–4 gm) in our study. This observation is in consonance with earlier reports, which suggest that increased hull thickness enhances seed weight but reduces oil content (Ranga Rao et al., 1977; Dajue and Mündel, 1996). Safflower oil has a desirable fatty acid composition. High linoleic lines of safflower are favored for animal feed and in the paint and varnish industry (Knowles, 1989; Bergman et al., 2001) while high oleic lines are nutritionally desirable because of its hypo-cholesterolemic effect and greater oxidative stability (Fuller et al., 1967). A high oleic line of Indian origin (Knowles and Bill, 1964) was effectively utilized in various safflower breeding programs in the USA (Mündel and Bergman, 2009). The safflower collection used in this study contained 17 accessions with high oleic acid content and 15 accessions with high linoleic acid content (Table 1). Accessions with desirable traits identified in the present study could be incorporated in breeding programs for crop improvement.

### Assessment of regional gene pools based on UPGMA analysis of phenotypic data

Accessions from the Indian Subcontinent and American region were distributed in all the five clusters (Figure 1) suggesting that they harbor maximum phenotypic diversity for the studied traits. Knowles (1969), based on morphological analysis of accessions from the Indian subcontinent, reported them as a uniform assemblage resulting from a single introduction. In contrast, our assessment indicates that accessions from the Indian subcontinent are phenotypically diverse. Morphological diversity among accessions from the Indian subcontinent was reported in earlier studies (Kupsow, 1932; Chavan, 1961; Hanelt, 1961). Indian accessions have also been shown to harbor significant genetic diversity (Kumar et al., 2015). The American germplasm was found to be phenotypically diverse in the current study but was genetically conserved (Kumar et al., 2015). Near East and Iran-Afghanistan accessions clustered together based on phenotypic data, supporting our earlier proposal of considering them as a single gene pool (Kumar et al., 2015). Accessions from European region were distributed in several clusters based on phenotypic data similar to the observation obtained through molecular data analysis. Interestingly, accessions from Far East, Turkey and Egyptian region were present in all the clusters although they exhibited low genetic diversity (Kumar et al., 2015). These results indicate that UPGMA analysis based on phenotypic data alone is unable to accurately define the genetic relationships among safflower accessions.

### Composite core collections effectively capture the global molecular, phenotypic, and geographical variability of the crop

In recent years, increased availability of molecular resources has enabled their utilization in development of core collections in crop species (Belaj et al., 2012; El Bakkali et al., 2013) but until now, no such attempts have been made in safflower. Use of molecular markers for development of core collections is advantageous as they reflect diversity at the DNA level as opposed to morphological markers wherein different genotypes might show similar phenotypic traits due to environmental effects. Additionally, molecular markers are more effective in identifying and minimizing redundancy. Several studies have emphasized on use of maximization (M) strategy for development of highly robust core collections (Bataillon et al., 1996; McKhann et al., 2004). The M strategy retains maximum number of alleles at each locus and is considered as the most powerful approach for maintaining diverse alleles (Schoen and Brown, 1993). MSTRAT and POWERCORE programs have been successfully used for construction of core collection in various plant species such as grapes, olive and sesame (Le Cunff et al., 2008; Belaj et al., 2012; Zhang et al., 2012). A combination of molecular markers and maximization (M) strategy has been utilized for the first time in our study for construction of a core collection in safflower.

Earlier studies reported significant GE interactions in safflower and emphasized on multi-location and multi-seasonal trials to evaluate heritability of characters for their effective utilization in breeding programs (Singh et al., 2004; Mahasi et al., 2006). In our study, MANOVA analysis indicated prominent GE interactions (Table 4). Therefore, seasonal datasets were treated independently for developing core collections. The six core collections thus generated, efficiently captured the entire range of trait variability but failed to include complete genetic diversity represented in 19 clusters derived earlier (Kumar et al., 2015) through Bayesian analysis. Additionally, many accessions were common between different core collections. For example, in core collections developed using POWERCORE, 10 accessions were common between CC1 (marker-based) and CC2/CC3 (phenotype-based). Only 4 accessions were unique to CC1 while 16 and 17 accessions were unique to CC2 and CC3, respectively. In MSTRAT-derived core collections, 19 accessions were common between CC4 (marker-based) and CC5/CC6 (phenotype-based). The number of accessions unique to CC4, CC5, and CC6 were 7, 28, and 35, respectively. The presence of common accessions between core collections derived using different types of data indicates an overlap in genetic and phenotypic components of the studied accessions. These accessions represent a subset of genotypes that are highly diverse at both molecular and phenotypic level.

The core collections developed using each program were merged to derive a more robust and non-redundant composite core collection (CartC1 by POWERCORE and CartC2 by MSTRAT). The vast phenotypic diversity of the initial collection was retained in both collections. Accessions with desirable agronomic traits and extreme phenotypes, which were present in very low numbers in the entire collection and scattered in the initial core collections were captured in the composite core collections (Table 8). Both the composite core collections provided comprehensive coverage of allelic diversity and had representation from all the 19 BAPS clusters identified earlier for safflower (Kumar et al., 2015; Table 7). Evaluation indices (MD%, VD%, VR%, CR%, *I, H*) for CartC1 and CartC2 were comparable and reflect their effectiveness in capturing diversity of the crop (Table 6). Our approach of deriving independent core collections from molecular and phenotypic data and their subsequent merger to create composite core collections avoided trade-off between the diversity captured using the molecular and phenotypic data sets.

Geographical distribution influences the extent of genetic variability of a species. The effect is more prominently seen in case of in-breeding species (Rao and Hodgkin, 2002). Geographical patterning is evident in safflower which is highly self-pollinating in nature and is grown in different agro-climatic regions across the world (Knowles, 1969; Ashri, 1975; Chapman et al., 2010). The two composite core collections showed minor variations in representation of the 10 regional gene pools. CartC1 included 8 regional pools excluding Sudan and Kenya while CartC2 contained representation from all the 10 regional gene pools (Table 5). Similar to the entire collection, both CartC1 and CartC2 showed predominance of accessions from Indian subcontinent and America accounting for ~50% of the total entries. In contrast, the earlier core collection developed by Johnson et al. (1993) had a major proportion of accessions (~46%) from the Mediterranean region and South-West Asia while the core collection derived by Dwivedi et al. (2005) consisted of ~78% accessions from South and South-East Asia.

The number of accessions in a core collection is an important factor determining its effective utilization (Brown and Spillane, 1999). The core collections developed earlier for safflower consisted of 210 accessions (Johnson et al., 1993) and 570 accessions (Dwivedi et al., 2005) while the composite core collections developed in the present study are comparatively smaller with 57 (CartC1) and 106 (CartC2) accessions. The larger size of the core collections developed in earlier studies could be due to the larger number of accessions in their initial germplasm collection. However, the advantage of the present study is that the initial collection used for development of composite core collections has been characterized extensively for both molecular and phenotypic diversity and the generated core collections have therefore effectively captured the global genetic and phenotypic diversity of the crop. Additionally, CartC1 has better utility value in comparison to CartC2 due to its smaller size and comparable diversity.

The present study is the first attempt where molecular diversity data has been used in conjunction with phenotypic data and geographical distribution to develop core collections in safflower. The small size of the composite core collections would be advantageous for field studies and association mapping. These collections will provide access to genetically diverse and agronomically important germplasm that would be useful in widening the genetic base of the crop and facilitate characterization of genetic determinants of trait variability. This information can be used to design more effective breeding programs to increase the global utility of safflower as an oilseed crop.

## Author contributions

AJ, SG conceived and designed the experiments. SK, HA performed the experiments, analyzed the data. MV helped in collection of phenotypic data. AR provided necessary support in the statistical analysis. SK, HA, SG, AJ wrote the manuscript. MA, AK, AJ, SG provided facilities for completion of experiments and reviewed the manuscript.

### Conflict of interest statement

The authors declare that the research was conducted in the absence of any commercial or financial relationships that could be construed as a potential conflict of interest.
