# Supplementary material for: Utilization of Molecular, Phenotypic, and Geographical Diversity to Develop Compact Composite Core Collection in the Oilseed Crop, Safflower (Carthamus tinctorius L.) through Maximization Strategy
Source: Front Plant Sci. 2016 Oct 19;7:1554. doi: 10.3389/fpls.2016.01554 (PMC5069285; doi:10.3389/fpls.2016.01554)
Supplement: Supplementary file 1 [file Table1.PDF]

Supplementary Table 1: Ranges, means and variances for the entire and core collections (season 2011-2012) derived using POWERCORE and MSTRAT.

Phenotypic Traits	Entire collection (Season 2011-2012)			CC 2 (POWERCORE)			CC 5 (MSTRAT)		
	Range	Mean	Variance	Range	Mean	Variance	Range	Mean	Variance
Oil content (%)	16-50	31	20.2	19-50	32	51.5	16-50	32	48.9
Oleic acid (%)	9-82	19	161.2	11-77	32	517.5	11-79	32	605.9
Linoleic acid (%)	13-87	71	151.7	13-87	59	493.3	13-87	59	574.5
Seed weight (gm)	1-8	5	2.0	1-8	4	3.0	1-8	4	3.2
Plant height (cm)	94-226	154	578.3	111-226	158	654.5	97-226	154	913.3
Number of heads per plant	11-203	74	1508	19-203	94	4607.9	19-194	88	3320.1
Number of primary branches	5-33	14	25.5	6-33	17	54.7	6-33	17	49.2
Days to 50%	119-160	141	44.0	123-159	141	76.1	119-	140	126.7

**flowering**							160		
