# Supplementary material for: Utilization of Molecular, Phenotypic, and Geographical Diversity to Develop Compact Composite Core Collection in the Oilseed Crop, Safflower (Carthamus tinctorius L.) through Maximization Strategy
Source: Front Plant Sci. 2016 Oct 19;7:1554. doi: 10.3389/fpls.2016.01554 (PMC5069285; doi:10.3389/fpls.2016.01554)
Supplement: Supplementary file 2 [file Table2.PDF]

Supplementary Table 2. Ranges, means and variances for the entire and core collections (season 2012-2013) derived using POWERCORE and MSTRAT

Phenotypic Traits	Entire collection (Season 2012-2013)			CC 3 (POWERCORE)			CC 6 (MSTRAT)		
	Range	Mean	Variance	Range	Mean	Variance	Range	Mean	Variance
**Oil content (%)**	15-47	30	19.6	15-47	31	39.1	15-45	30	33.2
**Oleic acid (%)**	9-82	19	161.2	11-79	29	511.9	11-77	23	261.5
**Linoleic acid (%)**	13-87	71	151.7	15-87	61	466.5	13-87	67	251.1
**Seed weight (gm)**	2-8	4	1.6	2-8	4	2.2	2-8	4	1.8
**Plant height (cm)**	73-211	128	567.5	80-203	134	1017.3	80-203	134	801.2
**Number of heads per plant**	9-189	71	1110.8	22-189	82	2190.4	22-189	79	1682.2
**Number of primary branches**	4-34	14	24.4	6-34	16	46.3	6-32	16	37.4
**Days to 50%**	137-182	160	51.1	137-182	169	111.4	137-182	116	138.9

**flowering**									
