# Supplementary material for: Utilization of Molecular, Phenotypic, and Geographical Diversity to Develop Compact Composite Core Collection in the Oilseed Crop, Safflower (Carthamus tinctorius L.) through Maximization Strategy
Source: Front Plant Sci. 2016 Oct 19;7:1554. doi: 10.3389/fpls.2016.01554 (PMC5069285; doi:10.3389/fpls.2016.01554)

Supplementary Figure 1. Trait distribution curve for three quantitative traits for two growing seasons.

(A, B) Plant height (cm); (C, D) Days to 50% flowering; (E, F) Oil content (%) for respective seasons. Boxes represents the tails of the distribution curve

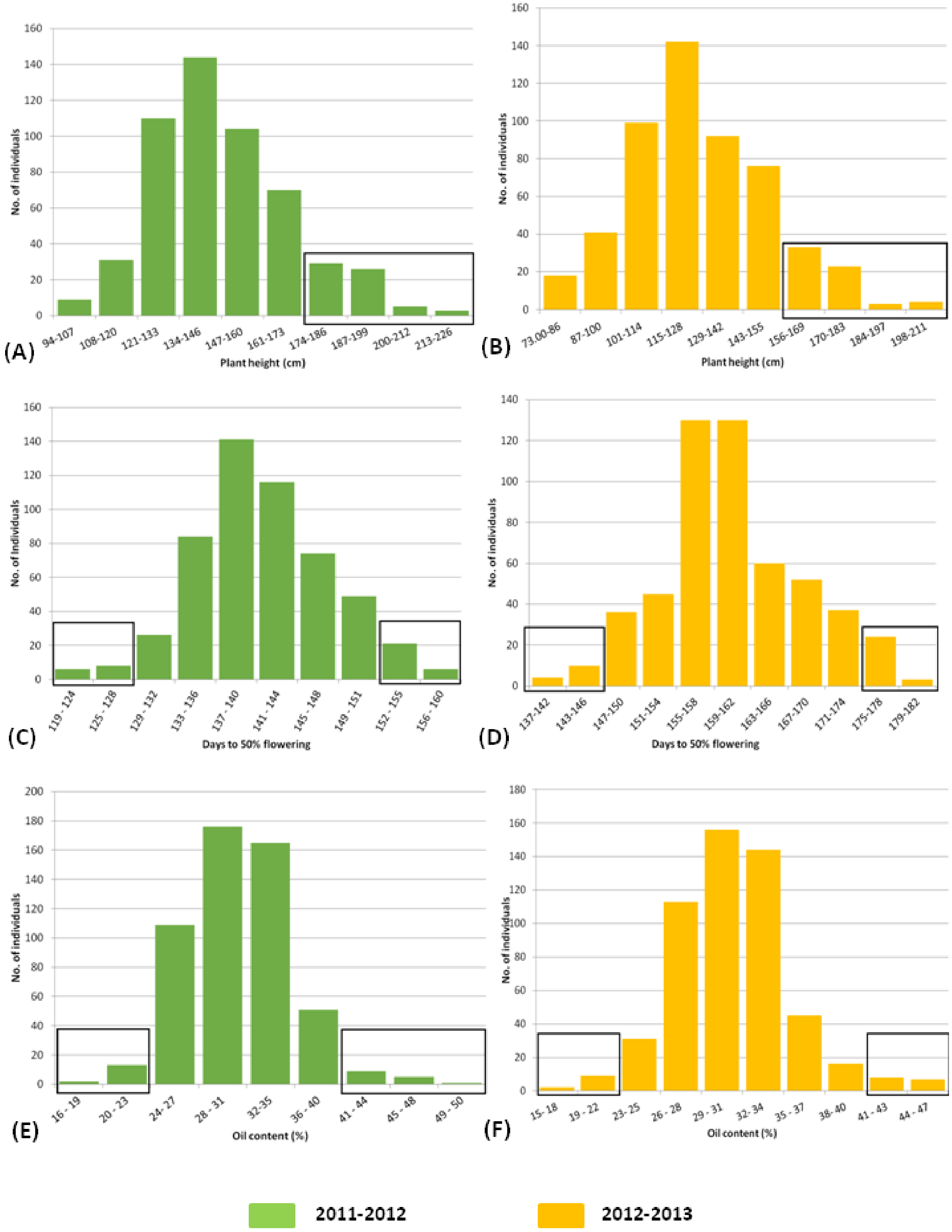

Supplement: Supplementary file 5 [file Image1.PDF]
